# The development and pilot testing of a shared decision-making encounter aid for weight management decisions

**DOI:** 10.1016/j.obpill.2026.100309

**Published:** 2026-07-31

**Authors:** Abd Moain Abu Dabrh, Khushboo S. Gala, Jiakun Yu, Emily T. Najlis, Max Plebani, Maria Daniela Hurtado Andrade, Iris G. Hargraves, Jennifer E. Clark, Michael L. Ganz, Gretchen E. Ames, Enrique F. Elli, Richard O. White, Sally-Ann L. Pantin, Andres Acosta, Juan P. Brito

**Affiliations:** a Division of Nephrology and Hypertension, Department of Medicine, Mayo Clinic, Jacksonville, FL, 32224, USA; b Division of Gastroenterology and Hepatology, Mayo Clinic, Rochester, MN, 59905, USA; c Department of Psychological and Brain Sciences, University of North Florida, Jacksonville, FL, 32224, USA; d Division of Endocrinology, Diabetes and Metabolism, Mayo Clinic, Rochester, MN, 59905, USA; e Knowledge and Evaluation Research Unit, Mayo Clinic, Rochester, MN, 59905, USA; f Division of Endocrinology, Diabetes, Metabolism, and Nutrition, Department of Medicine, Mayo Clinic, Rochester, MN, 59905, USA; g Operations and Administrative Services, Mayo Clinic, Jacksonville, 32224, USA; h Department of Psychiatry and Psychology, Mayo Clinic, Jacksonville, FL, 32224, USA; i Department of Surgery, Mayo Clinic, Jacksonville, FL, 32224, USA; j Division of Community Internal Medicine, Department of Medicine, Mayo Clinic, Jacksonville, FL, 32224, USA; k Department of Family Medicine, Mayo Clinic, Jacksonville, FL, 32224, USA; l Center for Clinical and Translational Science, Mayo Clinic, Rochester, MN, 59905, USA

**Keywords:** Weight management, Obesity, Decision aid, Shared-decision making, Patient-centered care, Pilot study

## Abstract

**Background:**

Obesity management involves multiple treatment options with differing benefits, risks, costs, and burdens, making these decisions highly preference sensitive. Shared decision-making (SDM) is recommended in obesity care, and yet practical tools to support real-time SDM during clinical encounters remain limited. We developed and pilot-tested a point-of-care decision aid, Weight Management Choice, to support structured discussions about weight management options.

**Methods:**

This was a two-phase study combining a human-centered design process with a subsequent single-arm observational pilot study conducted in outpatient clinics at two Mayo Clinic sites. The design phase involved evidence review, observation of clinical encounters, iterative prototyping, and stakeholder-informed field testing. The pilot phase then evaluated the resulting tool in routine encounters between adults considering weight management options and participating clinicians. Post-encounter assessments included the SURE test, the 9-item Shared Decision Making Questionnaire (SDM-Q-9), and technology acceptance instrument assessing perceived usefulness (PU) and perceived ease of use (PEOU).

**Results:**

Five clinicians were enrolled, four completed at least one assessment. Of 40 patients enrolled, 29 completed at least one patient-reported assessment (median age, 43 years; 75.9% female). Among patients completing the SURE test (n = 27), the mean score was 3.81 (SD 0.77) on a 0–4 scale. Among those completing the SDM-Q-9 (n = 29), the mean score was 75.0 (SD 18.2) on a 0–100 scale. Patients completing the technology acceptance instrument (n = 26) reported mean PU and PEOU scores of 27.2 (SD 4.52) and 27.8 (SD 3.93), respectively. Across 33 clinician encounter-level assessments, corresponding mean scores were 27.5 (SD 2.74) and 29.1 (SD 2.74) on 5–35 scales.

**Conclusions:**

The Weight Management Choice aid was perceived as feasible and had favorable immediate post-encounter ratings of shared decision-making, decisional conflict, and perceived usability. Future research should evaluate its impact on longer-term decision quality, treatment adherence, and clinical outcomes across diverse care settings.

## Graphical abstract

**Figure:**
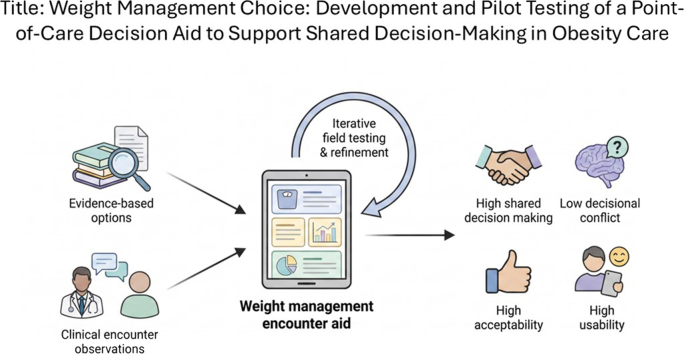

## 1 Introduction

Obesity is a complex, chronic, heterogenous and relapsing disease characterized by excess or dysfunctional adiposity that impairs health and well-being [1]. Its prevalence continues to rise globally and is a major driver of cardiometabolic conditions, including type 2 diabetes, cardiovascular disease, and obstructive sleep apnea, contributing substantially to healthcare burden and costs [2,3].

Management of obesity has evolved to include a spectrum of options — lifestyle interventions, pharmacotherapy, and metabolic procedures — each with distinct benefits, risks, costs, and treatment burdens [2,4,5]. These decisions are inherently preference-sensitive and shaped by patients’ goals, capacity, lived context, and access to resources [6,7]. However, clinical encounters often do not fully support structured deliberation across these trade-offs. Time constraints, variable clinician familiarity with rapidly evolving therapies, and the absence of practical decisional support tools may limit consistent implementation of patient-centered care [8,9]. Emerging digital and technology-enabled approaches have sought to address these gaps, although their integration into routine clinical workflows remains variable and often limited by complexity and implementation challenges. Additionally, weight stigma and implicit bias may further hinder open dialogue and engagement, negatively affecting care experiences and outcomes [10].

Shared decision-making (SDM) is increasingly recommended by major guidelines as a core component of high-quality clinical care [11,12], including obesity care [4,13,14], particularly when multiple reasonable treatment pathways exist [11,12,15]. SDM involves collaboration between patients and clinicians to integrate the best available evidence with patient values, preferences, and contextual factors [16,17]. Despite its endorsement, SDM remains inconsistently operationalized in routine practice, in part due to limited availability of tools designed for real-time use during clinical encounters [8,18].

Decision aids have been developed to support SDM by presenting evidence-based options and facilitating preference elicitation, and have been shown to improve knowledge, reduce decisional conflict, and enhance patient involvement [8]. However, many existing tools are lengthy, difficult to integrate into workflow, or not tailored to the complexity of obesity management [9,18]. Encounter-based decision aids — brief, structured tools designed for use during the clinical visit — offer a pragmatic alternative by supporting focused, patient-centered dialogue without substantially increasing visit burden. Prior work has demonstrated the feasibility, acceptability, and impact of such tools across chronic conditions, but their application in obesity care remains limited [19].

The aim of this study was to develop and pilot-test a point-of-care SDM decision aid, Weight Management Choice, to support discussions of obesity treatment options in adults. Using a human-centered design approach, we integrated characterization of usual communication and decision-making patterns with iterative, stakeholder-informed prototyping and field testing to develop the tool and conducted a proof-of-concept evaluation of its usability, acceptability, and patient-reported decision-making experience.

## 2 Methods

### 2.1 Study design

We conducted a two-phase study comprising a human-centered design and development phase followed by a proof-of-concept, single-arm observational pilot study at two Mayo Clinic outpatient sites; one in Rochester, MN, and one in Jacksonville, FL. The design phase involved evidence review, observation of clinical encounters, iterative prototyping, and stakeholder-informed field testing. The pilot phase then evaluated the resulting tool in routine encounters between adults considering weight management options and participating clinicians. Following each patient-clinician encounter, patients and clinicians completed post-visit assessments evaluating usability, acceptability, and patient-reported decision-making experience. All outcomes were assessed immediately after the encounter, without longitudinal follow-up.

### 2.2 Setting and participants

Participants were recruited using purposive sampling from outpatient clinics at the Department of Medicine at Mayo Clinic Rochester, MN, and at Mayo Clinic Jacksonville, FL practices. Eligible patients were adults (≥18 years) engaged in discussions about weight management options. Clinicians were healthcare professionals involved in obesity care. Recruitment occurred through referrals from the practicing clinicians at the outpatient practices, and all participants provided informed consent.

### 2.3 Development and field testing of Weight Management Choice

#### 2.3.1 Evidence synthesis and evaluation of usual practice

We developed a decision aid (Weight Management Choice) using a human-centered design approach informed by prior work. A multidisciplinary team of clinicians, shared decision-making researchers, and design experts led an iterative process that included (1) review of clinical evidence, (2) evaluation of usual clinical practice, (3) prototype development, and (4) field testing with iterative refinement. We synthesized the best available comparative effectiveness evidence across weight management interventions, drawing primarily from randomized clinical trials, systematic reviews, and network meta-analyses comparing lifestyle interventions, pharmacologic therapies, and bariatric procedures. The evidence was summarized in an evidence grid emphasizing patient-relevant outcomes, including expected weight loss, side effects, added health benefits, treatment burden, and cost.

#### 2.3.2 Prototype development and iterative refinement

To evaluate usual practice, we observed and, when feasible, recorded clinical encounters at Mayo Clinic Rochester and conducted post-encounter interviews with patients and clinicians to identify communication patterns, informational needs, and barriers to engagement. These insights informed the development of an initial prototype designed to support real-time comparison of options and elicitation of patient preferences (see prototype in Supplementary Materials Figs. 1–5). The prototype was then tested in routine clinical encounters and refined iteratively through cognitive walkthroughs, usability testing, and stakeholder feedback. Revisions focused on improving clarity, balance of information, and feasibility for integration into clinical workflows. Iteration continued until the tool consistently supported structured, patient-centered conversations in which patients engaged in comparing options and expressing preferences aligned with their context and goals.

### 2.4 Observational pilot evaluation and outcomes

Patient-reported measures included decisional conflict using the SURE test (Decisional Conflict Scale short form), shared decision-making using the nine-item Shared Decision Making Questionnaire (SDM-Q-9), and technology acceptance using an Innovation and Technology Management-based instrument (Technology Acceptance Model or TAM) assessing perceived usefulness (PU) and perceived ease of use (PEOU). Clinicians also completed the same ITM-based instrument (TAM) to assess PU and PEOU for usability and integration into clinical workflows. The SURE test is a four-item questionnaire with yes/no responses, yielding a total score ranging from 0 to 4; higher scores indicate lower decisional conflict. The SDM-Q-9 is a nine-item questionnaire rated on a 6-point Likert scale (1 = completely disagree to 6 = completely agree), with total scores linearly transformed to a 0–100 scale, where higher scores reflect greater levels of shared decision-making. TAM scores were calculated as summed Likert-scale responses for each domain (PU and PEOU), based on 5 items per domain rated on a 7-point Likert scale (1 = strongly disagree to 7 = strongly agree), yielding domain scores ranging from 5 to 35, with higher scores indicating greater perceived usefulness and ease of use. TAM items capture perceived usefulness and ease of use, including perceived effects on communication and decision-making, and do not represent objective measures of clinical outcomes or decision quality.

### 2.5 Ethical considerations

The study was approved by the Mayo Clinic Institutional Review Board (Protocol No. 18–003484). This observational pilot study is not subject to ClinicalTrials.gov registration. All participants provided their informed consent prior to participation.

### 2.6 Statistical analysis

Quantitative data were summarized using descriptive statistics, including means and standard deviations for continuous variables. TAM domain scores (PU and PEOU) were summarized separately for patients and clinicians, with item-level responses aggregated without weighting.

Decisional conflict (SURE) and SDM-Q-9 scores were summarized descriptively for patients. Given the exploratory nature of this pilot study, analyses focused on descriptive patterns rather than inferential comparisons between patients and clinicians. Analyses were conducted using available-case data without imputation, and denominators are reported for each outcome to reflect variable survey completion. Data were collated and managed using Microsoft Excel 2026 and analyzed using Python (version 3.12).

## 3 Results

### 3.1 The Weight Management Choice decision aid prototype development

**Fig. 1:**
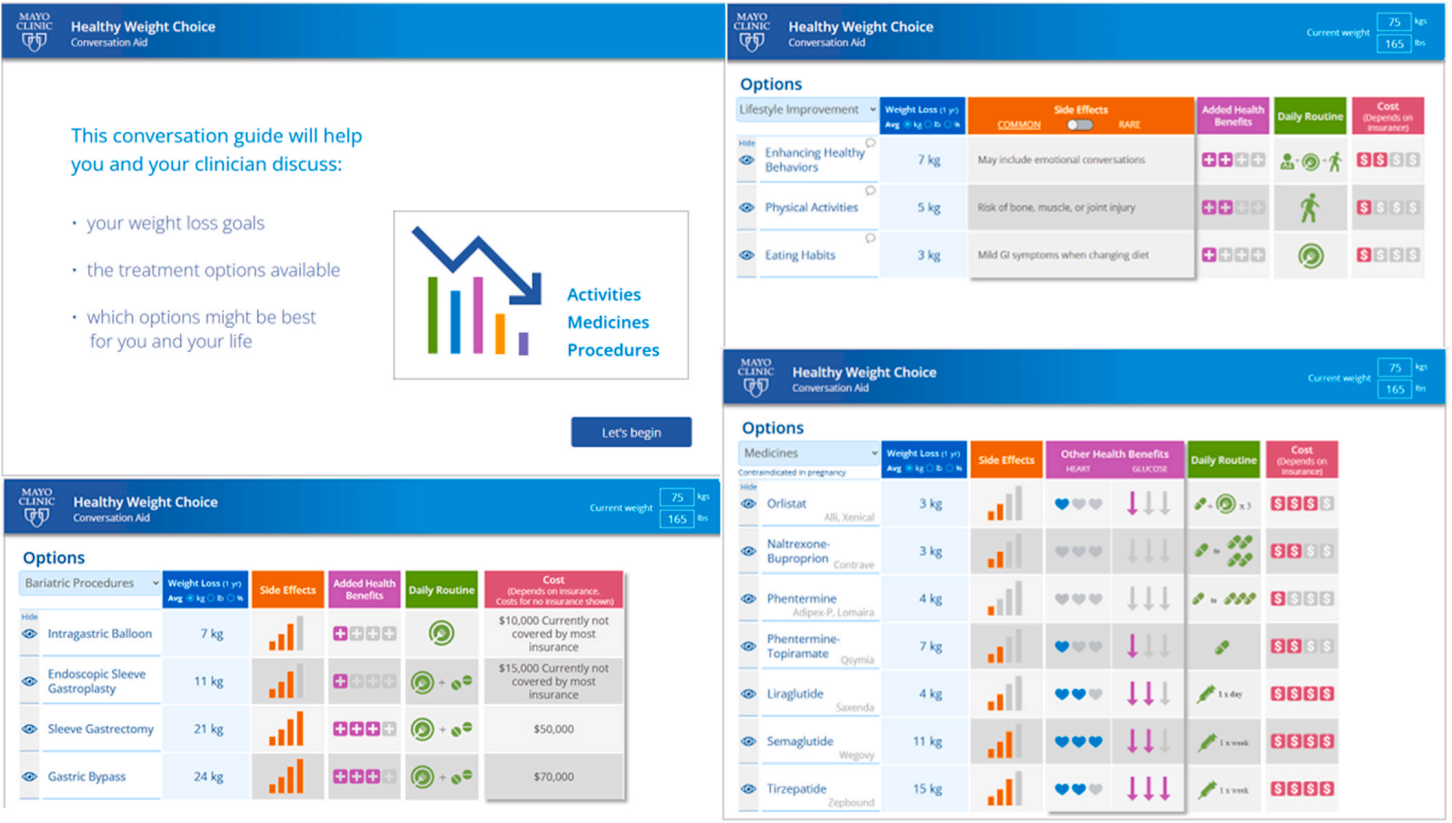
The weight management decision aid.

The Weight Management Choice aid was developed through an iterative process that integrated clinical evidence, observations from clinical encounters, and input from patients, clinicians, and other subject matter experts. The resulting prototype is a visual, encounter-based tool designed to support structured discussions about weight management by aligning treatment options with patient goals and preferences as shown in Fig. 1.

The tool opens with an introductory screen that frames the discussion's purpose, prompting patients and clinicians to consider weight-loss goals, review available treatment options, and identify which options best fit the patient's life context. It then transitions to an overview interface presenting three main categories of treatment options: lifestyle interventions (i.e., diet, exercise, and behavioral therapy), pharmacologic treatments, and bariatric procedures.

Treatment options are displayed in a comparative, tabular format that allows simultaneous review across key attributes, including expected weight loss at 1 year (in kilograms and pounds), side effects, added health benefits, daily routine requirements, and cost. The interface also allows entry of the patient's current weight to contextualize expected outcomes and support individualized discussion. Within each category, individual options can be explored in greater detail. Lifestyle interventions include behavioral therapy, exercise, Mediterranean diet, ketogenic diet, and combined approaches; pharmacologic options include orlistat, naltrexone-bupropion, phentermine, phentermine-topiramate, liraglutide, semaglutide, and tirzepatide; and procedural options include intragastric balloon, endoscopic sleeve gastroplasty, sleeve gastrectomy, and gastric bypass. Each option is presented consistently across the same set of attributes, facilitating comparison of trade-offs.

### 3.2 The Weight Management Choice decision aid pilot testing

A total of 40 patients were enrolled, of whom 29 completed at least one patient-reported measure and comprised the analytic sample. Completion of individual patient-reported measures ranged from 65.0% to 72.5%. Among the 29 patients included in the analysis, the median age was 43 years (IQR, 35–57), and 22 (75.9%) were female. Five clinicians were enrolled, of whom four completed at least one assessment. Clinicians completed 33 encounter-level assessments. Demographic characteristics of participants as summarized in Table 1.

**Table 1:** Baseline characteristics of included patients.

Variable	Included patients (N = 29)^a^
Age in yrs, median (IQR)	43 (35–57)
Gender, n (%)	
• *Male*	*6 (20.7%)*
• *Female*	*22 (75.9%)*
• *Non-binary*	*1 (3.4%)*
Ethnicity, n (%)	
• *White*	*25 (86.2%)*
• *Black or African American*	*3 (10.3%)*
• *Hispanic*	*1 (3.4%)*

a Of the 40 patients enrolled, 29 completed at least one survey and were included in the final analysis.

#### 3.2.1 Reported outcomes of pilot testing

**Fig. 2:**
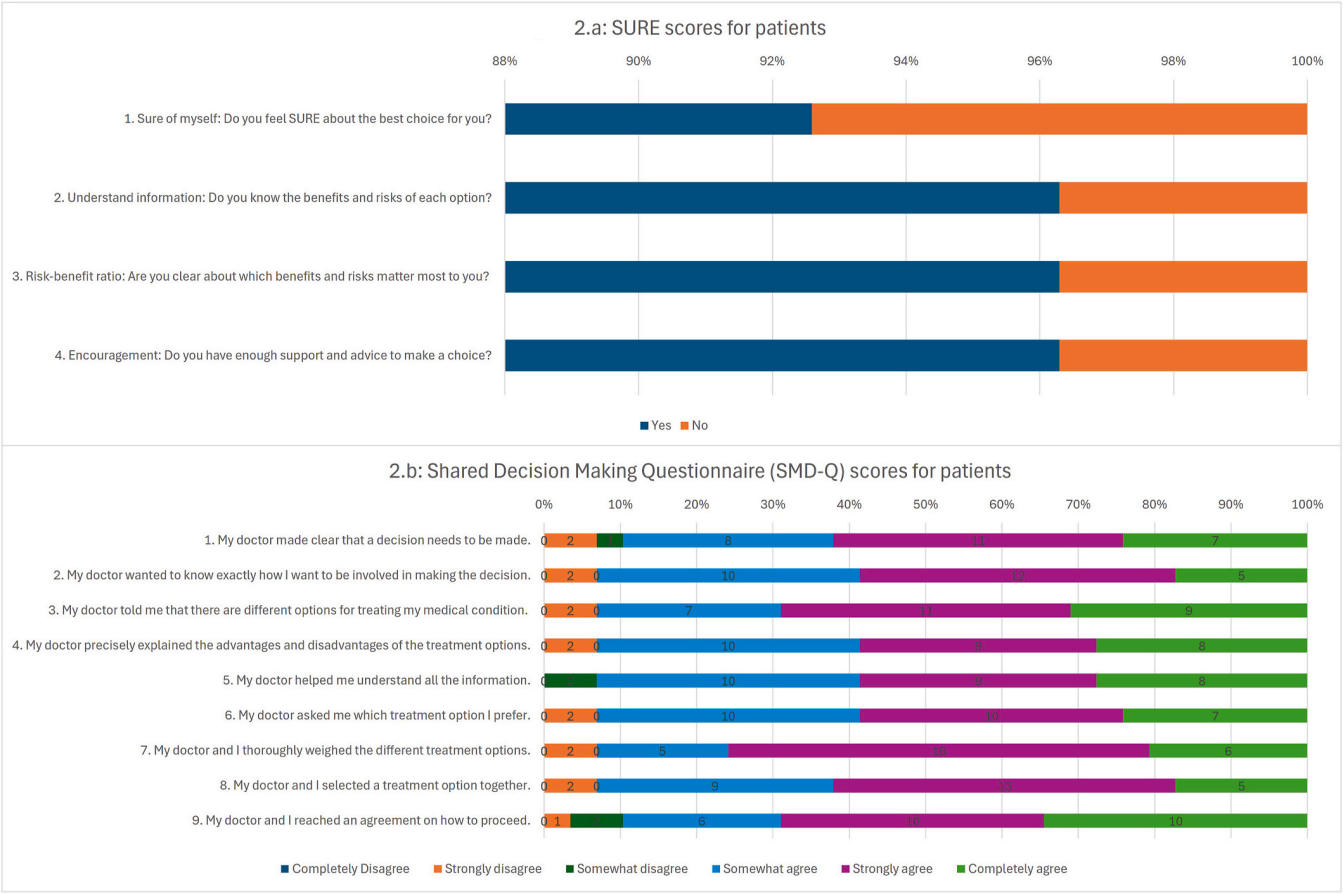
(a) SURE scores 27 patients. (b) Shared Decision Making Questionnaire scores for 29 patients.

Post-encounter evaluations reported favorable patient-reported decision-making experiences. Among patients who completed the SURE test (n = 27), the mean score was 3.81 (SD 0.77) on a 0–4 scale, with most participants reporting the maximum score of 4, consistent with low decisional conflict (Fig. 2a). Among patients who completed the SDM-Q-9 (n = 29), the mean score was 75.0 (SD 18.2) on the 0–100 scale, indicating favorable perceived involvement in shared decision-making (Fig. 2b).

**Fig. 3:**
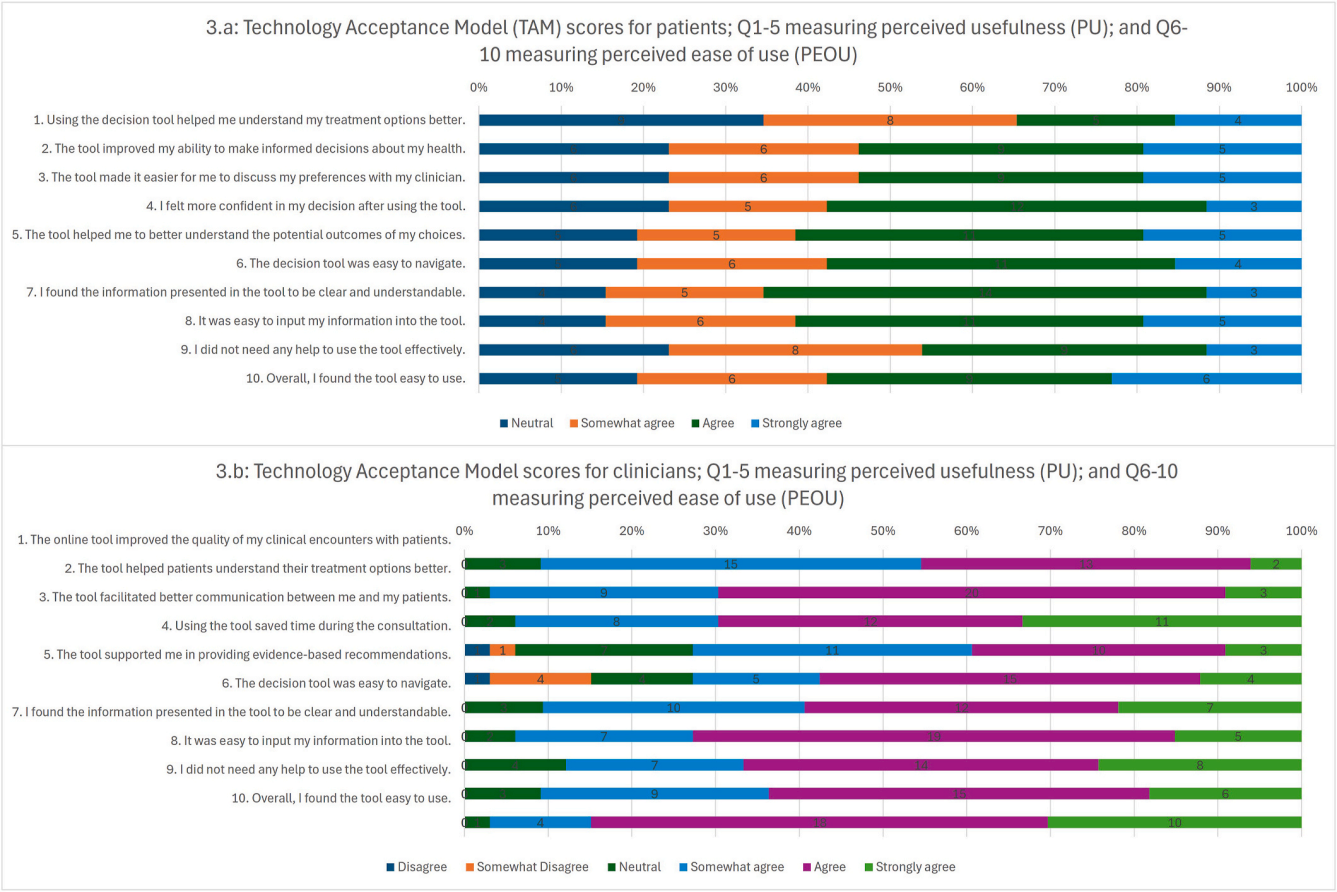
(a) Technology Acceptance Model (TAM) scores for 26 patients. (b) Technology Acceptance Model scores for 33 patient-clinician encounters.

Technology acceptance ratings were also favorable. Patients (n = 26) reported mean PU and PEOU scores of 27.2 (SD 4.52) and 27.8 (SD 3.93), respectively (Fig. 3a). Clinician responses across encounters (n = 33) showed mean PU and PEOU scores of 27.5 (SD 2.74) and 29.1 (SD 2.74), respectively (Fig. 3b). These findings suggest that both patients and clinicians perceived the decision aid as useful and easy to use during clinical encounters.

## 4 Discussion

We developed and field-tested a decision aid for weight management following a human-centered approach. In this pilot study, the tool was associated with favorable patient-reported shared decision-making, low decisional conflict, and high acceptability among participating patients and clinicians. These findings are consistent with the role of structured, point-of-care tools in facilitating patient-centered discussions in obesity care, a context characterized by complex, preference-sensitive decisions and substantial variability in treatment burden.

The observed ratings indicate that participants reported clarity, confidence, and engagement during clinical encounters. These findings are consistent with prior literature suggesting that encounter-based decision aids may support patient involvement and reduce decisional conflict in preference-sensitive conditions [8,19]. The moderately high perceived usefulness and ease of use reported by both patients and clinicians suggest the tool was acceptable for use within clinical encounters, addressing a common limitation of traditional decision aids, which are often too lengthy or impractical for real-time use [9].

However, these findings should be interpreted within the context of existing evidence. As a chronic, often progressive condition requiring multimodal management, obesity care should be individualized, with treatment intensity aligned with disease severity and patient context. Treatment selection extends beyond weight-loss magnitude alone and should incorporate durability, obesity-related complications, health-related quality of life, treatment burden, access, and long-term sustainability of care [20].​ As obesity treatment options expand, shared decision-making frameworks may help clinicians and patients align evidence-based options with patient preferences, values, and informed choice​ [21].​ While decision aids and structured SDM tools consistently improve proximal outcomes such as knowledge and decisional conflict, their effects on long-term behavioral and clinical outcomes remain variable [8,9]. In obesity care specifically, where sustained behavior change and adherence are critical, improving decision quality alone may be necessary but not sufficient to achieve meaningful long-term outcomes. These findings underscore the importance of embedding such tools within broader, personal capacity-aligned care models that address behavioral, social, and contextual determinants of health, recognizing that improving patient-reported decision-making experiences alone may be insufficient to influence long-term outcomes [6,7,16,22].

Our findings also have relevance in the context of emerging digitally enabled approaches, including artificial intelligence (AI), to obesity care. Recent reviews suggest that these tools may support engagement and short-term behavioral or metabolic outcomes, but the evidence remains heterogeneous and limited by short follow-up, variable intervention quality, and unresolved concerns about standardization, equity, explainability, and oversight [23,24]. In contrast, brief human-centered decision aids offer a transparent, low-complexity approach to supporting patient-reported decision-making within the clinical encounter, without relying on autonomous or opaque digital systems [6,25]. Future research should also explore whether AI-enabled features can be integrated into decision aids to further tailor information delivery and support deliberation while maintaining transparency, clinician oversight, and patient-centered decision-making [23].

## 5 Strengths and limitations

There are some limitations that should be acknowledged in this study. This was a single-arm pilot study with a modest sample size and limited demographic representation, limiting generalizability and precluding causal inference. Outcomes were primarily self-reported and measured immediately post-encounter, which may overestimate effects because of social desirability or recall bias. The high SURE scores observed suggest a potential ceiling effect, limiting discrimination in decisional conflict measurement. Additionally, the study did not assess downstream outcomes such as treatment adherence, weight change, or longer-term patient-reported outcomes. Finally, the current prototype emphasizes expected weight-loss outcomes and does not fully account for baseline obesity-related complications or other patient-specific factors that increasingly inform individualized treatment selection.

However, our study has several strengths. First, it used a human-centered design approach, enabling iterative development grounded in real-world patient and clinician input and facilitating future refinement as evidence and treatment options evolve. Second, the decision aid was developed and evaluated within routine clinical encounters across two Mayo Clinic sites in Minnesota and Florida, enhancing ecological validity and relevance to practice. Third, the study incorporated validated patient-reported measures of decisional conflict and shared decision making, providing structured assessment of key proximal outcomes. Finally, inclusion of both patient and clinician perspectives on usability and acceptability provided a more comprehensive assessment of the tool's perceived feasibility and ease of use in clinical encounters.

## 6 Conclusion

A brief, encounter-based decision aid for weight management was perceived as feasible and had favorable immediate post-encounter ratings of shared decision-making, decisional conflict, and usability. By structuring discussions around evidence-based options, patient preferences, lived context, and clinical characteristics, such tools may support patient-reported decision-making experiences in weight management care. Future studies should evaluate their impact on longer-term decision quality, treatment adherence, consultation length, and clinical outcomes across larger and more diverse populations. As obesity therapies and evidence continue to evolve, future iterations of the decision aid should incorporate emerging treatment options and additional patient-specific factors to support increasingly individualized treatment decisions.

## Author contribution

The concept of the submission was by AMAD, KSG, AA, and JPB. Data Curation as well as statistical and qualitative analyses were performed by AMAD, JY, EN, MP, IH, JC, MG and SAP. First draft was written by AMAD, KSG, JY, JC. All co-authors, including KG, JY, EN, MP, MA, IH, JC, MG, GA, EFE, RW, SAP, and AA reviewed, edited, and approved the final submission for publication.

## Disclosures

Andres Acosta and Mayo Clinic are inventors of intellectual property licensed to Phenomix Sciences Inc. Andres Acosta served as a consultant in the last 5 years for Rhythm Pharmaceuticals, Gila Therapeutics, Amgen, General Mills, Boehringer Ingelheim, Currax Pharmaceuticals, Nestle, Bausch Health, RareDiseases; has received research support or contracts with Vivus Inc, Satiogen Pharmaceuticals, Boehringer Ingelheim, and Rhythm Pharmaceuticals. Acosta has received funding from National Institute of Health, Rhythm Pharmaceuticals, Boehringer Ingelheim, Regeneron, Terns, Novo Nordisk, Vivus Inc, Dairy Management Institute, and Phenomix Sciences in the last 3 years. The authors declare no other conflicts of interest.

## Ethical adherence and ethical review

The study was approved by the institutional review board (IRB) at Mayo Clinic. All participants provided informed consent prior to participation in accordance with The Code of Ethics of the World Medical Association (Declaration of Helsinki).

## Clinical takeaways

1.Developed and pilot-tested a brief, human-centered decision aid for weight management, using iterative input from both patients and clinicians to support shared decision-making during clinical encounters.
2.Patients reported high levels of shared decision-making, low decisional conflict, and strong engagement, indicating a positive decision-making experience during weight management consultations.
3.Both patients and clinicians rated the decision aid as useful, easy to use, and acceptable within routine clinical encounters.
4.Although findings support the feasibility and acceptability of the decision aid, the single-arm pilot design, small sample size, and lack of long-term outcome assessment limit conclusions about its impact on treatment adherence, weight loss, and clinical outcomes.

## Declaration of artificial intelligence (AI) and AI-assisted technologies

During preparation of this work, the authors conceived and wrote the original manuscript in full. After co-author review and input, AI-assisted technology was used only for language editing and readability improvements. The authors reviewed and edited the output as needed and took full responsibility for the publication's content. No AI tools were used to generate scientific content, analyze data, interpret results, or draw conclusions.

## Source of funding

This work was funded in part by the Mayo Clinic Florida Clinical Practice Committee and the Clinical Research Operations group of Mayo Clinic Florida.
