# Supplementary material for: The development and pilot testing of a shared decision-making encounter aid for weight management decisions
Source: Obes Pillars. 2026 Jul 31;19:100309. doi: 10.1016/j.obpill.2026.100309 (PMC13473964; doi:10.1016/j.obpill.2026.100309)
Supplement: Supplementary file 1 [file mmc1.docx]

Title: The Development and Pilot Testing of a Shared Decision-Making Encounter Aid

for Weight Management Decisions

Supplementary Materials

Figure 1: Weight Management Choice Conversation Aid - home page

Figure 2: Weight Management Choice Conversation Aid - Overview

Figure 3: Weight Management Choice Conversation Aid -Lifestyle Improvement

Figure 4: Weight Management Choice Conversation Aid -Medicines

Figure 5: Weight Management Choice Conversation Aid -Bariatric Procedures
